# A Drug-Sensitive Genetic Network Masks Fungi from the Immune System

**DOI:** 10.1371/journal.ppat.0020035

**Published:** 2006-04-28

**Authors:** Robert T Wheeler, Gerald R Fink

**Affiliations:** Whitehead Institute for Biomedical Research, Cambridge, Massachusetts, United States of America; Johns Hopkins University, United States of America

## Abstract

Fungal pathogens can be recognized by the immune system via their β-glucan, a potent proinflammatory molecule that is present at high levels but is predominantly buried beneath a mannoprotein coat and invisible to the host. To investigate the nature and significance of “masking” this molecule, we characterized the mechanism of masking and consequences of unmasking for immune recognition. We found that the underlying β-glucan in the cell wall of Candida albicans is unmasked by subinhibitory doses of the antifungal drug caspofungin, causing the exposed fungi to elicit a stronger immune response. Using a library of bakers' yeast *(Saccharomyces cerevisiae)* mutants, we uncovered a conserved genetic network that is required for concealing β-glucan from the immune system and limiting the host response. Perturbation of parts of this network in the pathogen C. albicans caused unmasking of its β-glucan, leading to increased β-glucan receptor-dependent elicitation of key proinflammatory cytokines from primary mouse macrophages. By creating an anti-inflammatory barrier to mask β-glucan, opportunistic fungi may promote commensal colonization and have an increased propensity for causing disease. Targeting the widely conserved gene network required for creating and maintaining this barrier may lead to novel broad-spectrum antimycotics.

## Synopsis

Opportunistic fungal pathogens such as Candida albicans often cause fatal infections in patients with a compromised immune system. Unfortunately, current drugs often fail to halt fungal disease, are ineffective against drug-resistant strains, and have severe side effects. Despite the clear clinical significance of fungal infections, it is still not understood how fungi are recognized by the immune system. *Candida* has high levels of the structural molecule β-glucan in its cell wall, but the majority of its β-glucan is masked by a mannoprotein coat and is therefore invisible to the immune system. Masking of β-glucan may be a fungal virulence factor, because exposed β-glucan provokes a proinflammatory response that is important for mounting an effective immune response against the fungus and clearing the infection. By surveying the genome of the model fungus Saccharomyces cerevisiae (bakers' yeast), the authors discovered a genetic network required for masking β-glucan from the immune system. Mutation of genes in this network in C. albicans caused unmasking of β-glucan and an increased immune response to the fungus. The authors also found that sublethal doses of the antifungal drug caspofungin cause unmasking and lead to a greater immune response. Drugs targeting this fungally conserved masking network may provide new tools to fight fungal infections.

## Introduction

**Figure 1:**
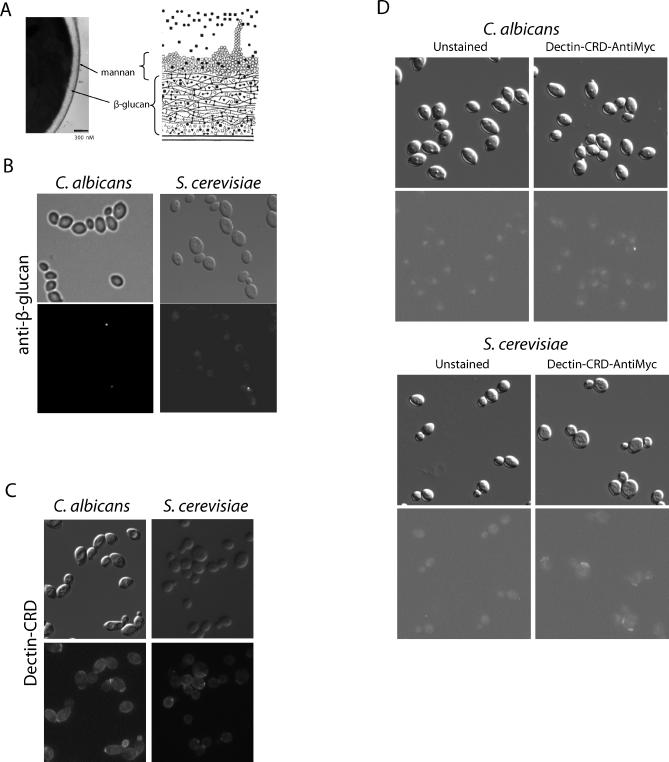
Fungal β-Glucan Is Buried in the Cell Wall and Largely Inaccessible (A) Transmission electron micrograph demonstrating layer structure of fungal cell wall (courtesy of C. Rondeau). The plasma membrane is tightly connected to a thick layer of β-glucan network. Mannoproteins are linked to β-glucan and protrude outside of this layer to make up a dense coat. Schematic adapted from [5]. (B–D) There is little β-glucan on live C. albicans or S. cerevisiae that is exposed and accessible to the anti-β-glucan antibody (B), the Dectin-CRD (C), or the Dectin-CRD-anti-Myc probe (D). The staining with anti-β-glucan and Dectin-CRD-anti-Myc is nearly indistinguishable, and is more specific than that with the directly labeled Dectin-CRD. The Dectin-CRD-anti-Myc has the same size as an antibody and the same specificity as Dectin-1. The difference in staining between the β-glucan-binding reagents (B and D versus C) is likely due to the size of the reagents (IgG has dimensions of approximately 95 Å × 171 Å [33], while the CRD of CD69, which is similar to the Dectin-1 CRD, has dimensions of 44 Å × 32 Å × 30 Å [34]) relative to the estimated pore size of the S. cerevisiae cell wall (58 Å [23]), and thus the monomeric Dectin-CRD likely has more access to smaller areas of exposed β-glucan.

The innate immune system recognizes microbes based on their pathogen-associated molecular patterns (PAMPs), which provoke pathogen-specific responses tailored to meet the challenge [1]. This ensures that parasites, viruses, bacteria, and fungi are each attacked appropriately. Fungi are opportunistic pathogens responsible for occasionally severe disease in individuals with systemic disease [2,3]. *Candida albicans,* clinically the leading fungal pathogen, is recognized predominantly by two PAMPs (β-glucan and mannan), which account by weight for over 90% of its cell wall [4,5]. The cell wall of these and other fungi is tiered, with an outer layer of mannoproteins covalently linked to an inner core of β-glucan (Figure 1A).

The inner β-glucan layer is an essential cell wall component targeted by fungicidal antibodies, immune receptors, and the echinocandin class of antifungal drugs. Recent work has shown that anti-β-glucan antibodies can directly kill fungi and assist in the clearance of fungal infection [6]. The β-glucan receptor Dectin-1 also recognizes fungi and mediates the innate immune system's proinflammatory response [7]. The importance of this layer is also underscored by the effectiveness of the echinocandins, which bind and inhibit β-glucan synthase to cause cell lysis [8].

The importance of this inner β-glucan layer for immune targeting belies an interesting paradox. Although it accounts for a major fraction of the cell wall, β-glucan is buried underneath a thin but dense mannan coat (Figure 1A) [9]. This tiered arrangement of the cell surface raises several important issues: How do the cells of the immune system recognize β-glucan if it is coated with mannoproteins? Furthermore, what fungal genes function to “mask” the β-glucan from the immune system?

In this study, we report the mechanisms and consequences of β-glucan masking and unmasking in the fungal-immune interaction. Growth of C. albicans in the presence of subinhibitory concentrations of the antifungal drug caspofungin exposes the normally masked β-glucan. We also define a conserved genetic network responsible for β-glucan masking in the genetically tractable, nonpathogenic model fungus Saccharomyces cerevisiae and confirm its requirement in the pathogen C. albicans. Exposure of β-glucan by drug treatment or mutation alters the way the fungi are recognized by immune cells, causing a β-glucan-dependent increase in the elicitation of tumor necrosis factor alpha (TNFα) from macrophages. For *C. albicans,* this unmasking transforms the fungi into cells that elicit a strong proinflammatory response. Thus, fungi camouflage the majority of their β-glucan from innate immune cells to lower the proinflammatory response to infection.

## Results

### The Cell Wall β-Glucan Is Masked in S. cerevisiae and C. albicans

Despite the high bulk levels of β-glucan throughout the fungal cell wall, C. albicans and laboratory strains of S. cerevisiae have little exposed β-glucan (Figure 1B–1D). The accessibility of β-glucan was measured in live fungal cells using three different reagents that colocalize: a monoclonal antibody directed against pure β-glucan [10], purified tagged Dectin-1 carbohydrate recognition domain (Dectin-CRD), and Dectin-CRD preincubated with fluorescently labeled antiepitope antibody (CRD-Myc; see Materials and Methods). Each of these three reagents interacts only weakly with intact cells of both C. albicans and laboratory strains of S. cerevisiae. Consistent with the biological relevance of masking of this proinflammatory molecule, intact cells of C. albicans and S. cerevisiae elicit little immune response when presented to macrophages.

In agreement with previous reports [11], the primary areas of glucan-inhibitable binding of Dectin-1 and the anti-β-glucan antibody are the sites of previous cell wall remodeling (i.e., bud scars, birth scars, and bud necks). Wild-type fungal cells have abundant β-glucan in their cell walls but present little β-glucan to the immune system, and this trace amount is restricted to the sites of cell division. As most of the cells in a culture are newly generated, they have completed only one division and would be less efficiently recognized by immune molecules. Thus, it seems that fungi have developed conserved pathways to conceal the potent proinflammatory molecule β-glucan from the immune system.

### Fungal β-Glucan Is Exposed by Subinhibitory Doses of Caspofungin

**Figure 2:**
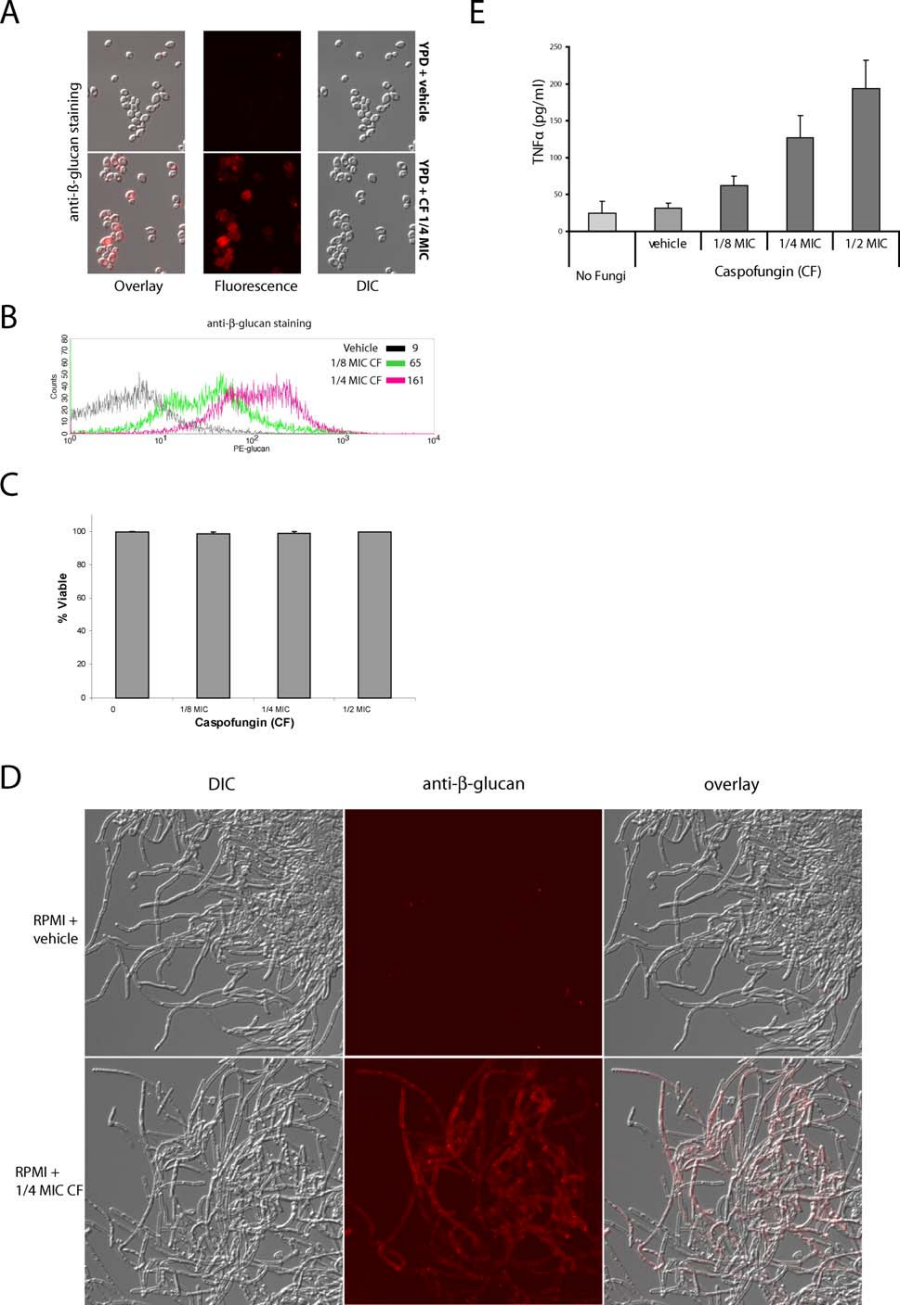
Subinhibitory Concentrations of the Antifungal Drug CF Cause Exposure of β-Glucan Wild-type C. albicans (CAF2) was grown overnight for ten generations in YPD medium, favoring yeast-form growth (A, B, C, and E) or RPMI medium, favoring hyphal growth (D). Cultures grown at one-quarter and one-eighth of the CF MIC_50_ were stained with anti-β-glucan antibody and Cy3-labeled secondary antibody (A and D) for visualization by microscopy or with PE-labeled secondary antibody (B) for FACS quantification. Mean fluorescence intensity (MFI) values were 9, 65, and 161, respectively, for no treatment, one-eighth the CF MIC_50_, and one-quarter the CF MIC_50_. Concurrently, cells were labeled briefly with propidium iodide to assess viability and visualized by epifluorescence microscopy or quantified by FACS (C). Cells grown overnight in YPD with or without CF were UV-inactivated and then exposed to BMDMs at a yeast:macrophage ratio of 10:1. Supernatants were taken at 6 h and assayed for TNFα (E).

We reasoned that antifungal drugs that specifically target cell-wall biosynthetic pathways would disrupt the intricate architecture of the cell wall and heighten β-glucan exposure, “unmasking” the cells through uncoating or cell wall disorganization. To examine this possibility, we grew wild-type C. albicans overnight in the presence of subinhibitory doses of caspofungin (CF), a potent echinocandin that is a recent addition to the antifungal arsenal [8]. Remarkably, at CF concentrations that permit normal growth rates the drug caused exposure of β-glucan on the C. albicans cell surface (Figure 2A and 2B). At these drug concentrations, the cells had 10×–30× greater reactivity with the anti-β-glucan antibody. It is important to note that the fungi grow at these drug concentrations without loss of viability (Figure 2C), suggesting that the increased β-glucan exposure is not due to generalized cell death. We also examined the effect of CF on cells grown in RPMI 1640, a culture medium that causes strong hyphal growth and β-glucan masking [11]. Subinhibitory doses of CF caused a dramatic increase in the exposure of β-glucan on hyphae grown in this media (Figure 2D). These cells also did not show increased cell death, as measured by microscopic examination.

Caspofungin inhibits the synthesis of β-glucan, which leads to lower bulk levels of β-glucan in the cell [8]; however, the inhibition of β-glucan biosynthesis also upsets the equilibrium required to maintain the normal tiered cell wall architecture [12]. This perturbation of the cell wall remodeling machinery can cause more exposure of β-glucan even in the presence of lower bulk levels of β-glucan. This fact also suggests that fungi have evolved a mechanism for masking β-glucan that is compromised by the drug.

### Caspofungin-Treated Cells Hyperelicit TNFα

Since β-glucan is the primary proinflammatory molecule on the fungal surface, we tested whether yeast unmasked by CF elicit larger proinflammatory responses from macrophages. This test requires inactivation of the fungi to prevent them from killing the macrophages before the end of the experiment. Previously used methods of inactivation (including heat, formaldehyde, and ethanol) artificially increase β-glucan exposure and lead to high levels of TNFα elicitation (Figure S1). We discovered that ultraviolet light (UV)-irradiated cells did not kill macrophages, retained an intact cell wall architecture, and elicited only low levels of TNFα (Figure S1). Therefore, we used UV-inactivated fungi for all subsequent experiments.

To test whether unmasked, CF-treated yeast elicit a greater proinflammatory response, we exposed CF-treated or -untreated (exposed or masked, respectively) C. albicans to primary bone marrow-derived macrophages (BMDMs) and assayed elicitation of the key proinflammatory cytokine TNFα. The CF-treated C. albicans elicited 3- to 4-fold higher levels of the key proinflammatory cytokine TNFα (Figure 2E) than did untreated *C. albicans,* which elicited undetectable levels of TNFα in this assay. Thus, subinhibitory doses of CF cause the pathogen to elicit a marked proinflammatory response.

### An Interconnected Genetic Network Is Required for β-Glucan Masking

To identify the system used by fungi to mask their β-glucan from the immune system, we screened a genome-wide library of knockout mutants in S. cerevisiae for increased β-glucan exposure. Because subinhibitory levels of CF are able to cause increased β-glucan exposure and altered recognition of the fungi without killing the fungi, we reasoned that this library of nonessential gene knockouts should identify genes that specifically perturb the genetic network required for β-glucan masking. Further, due to similarity in cell wall structure between these two fungi, the genes we identified in *Saccharomyces* could guide us to functionally equivalent genes in *Candida* that serve the same masking function.

**Figure 3:**
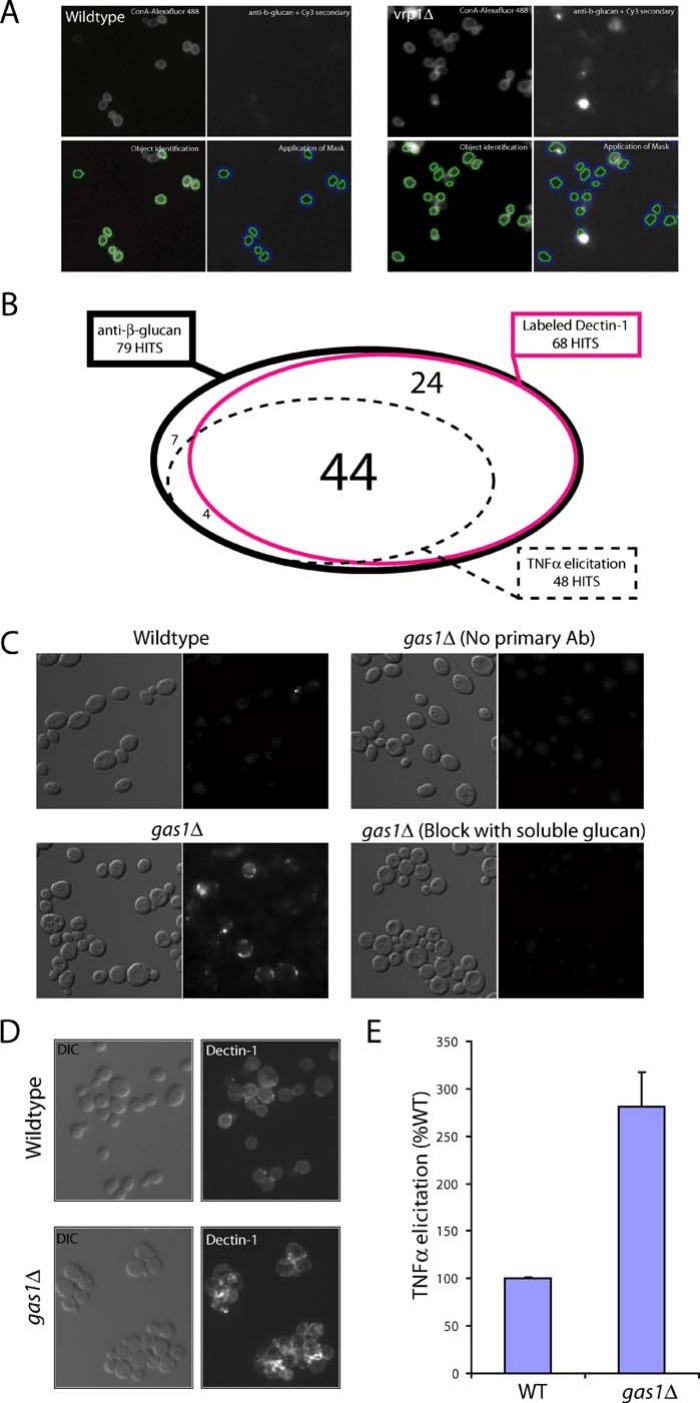
Automated Genome-Wide Screen for Increased β-Glucan Exposure We screened the genome-wide set of S. cerevisiae knockout mutants by staining with anti-β-glucan antibody and quantifying β-glucan exposure using the Cellomics system. (A) Images of yeast were taken at 20×, and object-finding software used the ConA fluorescence signal to identify cells (green outline); the software then applied a mask (blue outline) and quantified the average level of fluorescence from the anti-β-glucan channel for each cell. Wild-type yeast (left photomicrographs) show little or no fluorescence from the anti-β-glucan channel, whereas the unmasked *vrp1Δ* mutant shows high levels of fluorescence from this channel. (B) Most of the mutants found with increased β-glucan exposure also showed greater binding to Dectin-CRD and increased TNFα elicitation from RAW 264.7 macrophages. Of the 76 mutants identified with increased anti-β-glucan binding (encircled with the black line), 48 (encircled with the dotted line) hyperelicited TNFα from macrophages and 65 (encircled with the red line) showed increased binding to the labeled Dectin-CRD. Numbers within the black oval show the intersections of each of these groups (e.g., 44 had increased anti-β-glucan binding and increased Dectin-CRD binding and increased TNFα elicitation from macrophages). (C–E) To exemplify the methodology used, we chose the *gas1Δ* mutant, which has intermediate β-glucan exposure and TNFα elicitation. (C) Live wild-type (upper left image) or *gas1Δ* mutant (lower left image) S. cerevisiae were stained with anti-β-glucan antibody. To show specificity of the binding, *gas1Δ* cells were stained omitting primary antibody (upper right image) or antibody was preincubated in 100 μg/ml soluble glucan (laminarin) before and during the staining (lower right image). (D) Live wild-type or *gas1Δ* mutant cells were stained with Alexa Fluor-labeled Dectin-CRD. (E) Live wild-type or *gas1Δ* mutant cells were exposed to RAW264.7 macrophages at a ratio of 5:1 (yeast:macrophage), and supernatants were collected after 6 h and measured for TNFα levels.

Using automated microscopy, we systematically screened the entire library of approximately 4,800 S. cerevisiae mutants for strains with increased β-glucan exposure. This platform permits quantification of the fluorescence from high resolution pictures of the β-glucan- stained yeast. This method of quantification is exemplified in the comparison of the *vrp1Δ* mutant to wild-type (Figure 3A). Strains with levels of antibody binding greater than two standard deviations from the mean of each plate were identified, and independently created mutants were rescreened using the same protocol (see Materials and Methods).

Emphasizing the connection between β-glucan exposure and immune recognition, most of the mutants with greater exposed β-glucan also showed increased binding to the Dectin-1 β-glucan receptor (Figure 3B). The greater exposure of β-glucan and better binding to Dectin-CRD of these mutants raised the possibility that they would have altered interaction with cells of the immune system. Each S. cerevisiae mutant was exposed to RAW264.7 murine macrophages and tested for TNFα elicitation. A large percentage (48 of 76) of the unmasked mutants triggered a significantly stronger proinflammatory response than did the wild-type lab strain of *S. cerevisiae;* some elicited up to ten times the amount of TNFα as did wild-type (Figure 3B and Table S1). Taking *gas1Δ* as a representative mutant with an intermediate phenotype, we found increased binding of the anti-β-glucan antibody to this mutant, and this binding is blocked by soluble β-glucan (Figure 3C). The *gas1Δ* mutant also binds Dectin-CRD better and elicits a higher level of TNFα from macrophages (Figure 3D and 3E). The strong correlation between β-glucan exposure and increased TNFα elicitation suggests that β-glucan masking on the surface of *Saccharomyces* is a key factor in blocking the immune response to fungi.

**Figure 4:**
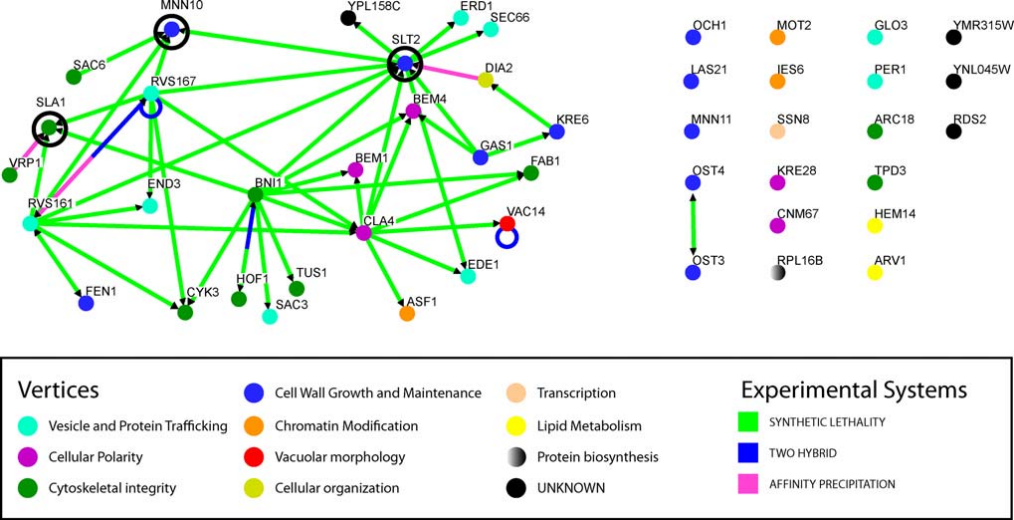
The Cell Wall Remodeling Network Required for β-Glucan Masking A map of physical and genetic interactions among β-glucan masking genes was made using the Osprey v1.2.0 visualization program (available at: http://biodata.mshri.on.ca/osprey/servlet/Index). All genes of hypereliciting mutants identified in the screen are represented. Lines connecting genes are colored to represent the nature of known interaction(s) between a pair of genes (i.e., synthetic lethality, two-hybrid, or coimmunoprecipitation). Vertices are colored to represent the cellular processes directed by the gene product, as annotated by the Yeast Proteome Database (available at http://www.proteome.com). Vertices of genes required for caspofungin resistance [15,16] are circled in black.

Most of the genes identified in our screen for the masking of β-glucan from the immune system fit under an umbrella of interconnected gene networks that regulate polarized cell wall remodeling (Figure 4): polarization of the actin cytoskeleton, polarized secretion of proteins and polysaccharides, and polarized endocytosis of unwanted byproducts [13,14]. The connections among these related pathways imply that coordination of polarized cell wall remodeling at sites of new cell wall addition is crucial for masking β-glucan. This interconnected network is sensitive to caspofungin. Three of the key hubs of the network *(SLT2, SLA1,* and *MNN10)* are required for caspofungin resistance [15,16]. Furthermore, the Slt2p mitogen-activated protein kinase is a key mediator of the caspofungin-induced stress response [16].

In addition, several genes required for mannosylation of cell wall proteins *(MNN10, MNN11, OCH1, OST3,* and *OST4)* are also required for masking of β-glucan and immune recognition. The connection between mannoprotein processing and β-glucan masking buttresses the idea that a dense coat of mannosylated cell wall proteins masks β-glucan from recognition. Apparently the increase in exposure is due to uncoating or disorganization of the cell wall rather than bulk changes in β-glucan levels, because in those hypereliciting mutants for which the bulk level of β-glucan is known, the vast majority of mutants (14 of 18) has unchanged or lower levels of both types of β-glucan (Table 1).

**Table 1:**
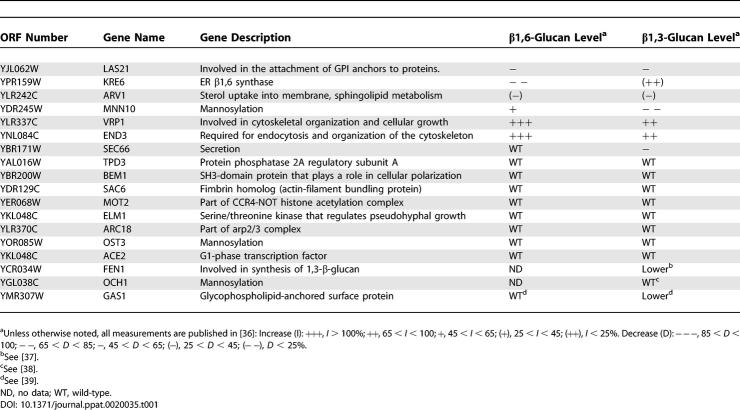
β-Glucan Exposure Does Not Correlate with Bulk Level in S. cerevisiae Mutants

As well as finding genes known to play a role in cell wall architecture (Figure S2), our screen identified many new genes that likely play roles in cell wall function (Figure 4). We identified four genes that encode global transcriptional regulatory proteins *(ASF1, IES6, MOT2,* and *SSN8)* and six genes with previously unassigned biological function *(DIA2, VAC14, RDS2, YMR315W, YNL045W,* and *YPL158C)* that were not expected a priori to be required for masking β-glucan or for recognition by the immune system. The stringency of our screen clearly implicates each of these genes in the cell wall remodeling process.

Several of the mutants with increased binding to anti-β-glucan antibody did not hyperelicit TNFα from macrophages. This could be due to different epitope specificity between antibody and the full-length receptor on the macrophage. To address this issue, we probed all of the “unmasked” mutants with a Dectin-CRD-anti-Myc probe (CRD-Myc) that colocalizes with Dectin-CRD and Dectin-1 (see Figure 1A). This CRD-Myc probe should have a similar size to anti-β-glucan and the same binding specificity as the Dectin-1 receptor. Only three of the 28 non-hyperelicitors showed even marginally increased binding to both Dectin-CRD and CRD-Myc *(gup1Δ, kre11Δ,* and *ylr111wΔ),* suggesting that differences in epitope recognition between the anti-β-glucan antibody and receptor account for a majority of the mutants that have greater anti-β-glucan binding but no hyperelicitation (Table S2).

Alternatively, different levels of mannoprotein could independently alter immune response and confound these results. To examine the surface mannoprotein structure, we quantified the level of exposed mannose on the surface of live yeast by incubating with the mannose-specific lectin concanavalin A (ConA). Although two mutants with known roles in mannosylation (i.e., *van1Δ, mnn2Δ*) showed reduced binding to ConA, we did not find a preponderance of mutants with reduced binding among our set of exposed mutants and, importantly, did not see any correlation between ConA binding and TNFα elicitation (Table S2). Therefore, under these conditions, overall levels of mannan on the surface do not appear to regulate TNFα elicitation independently of β-glucan recognition.

### The Genetic Pathway for β-Glucan Masking Is Conserved in C. albicans

**Figure 5:**
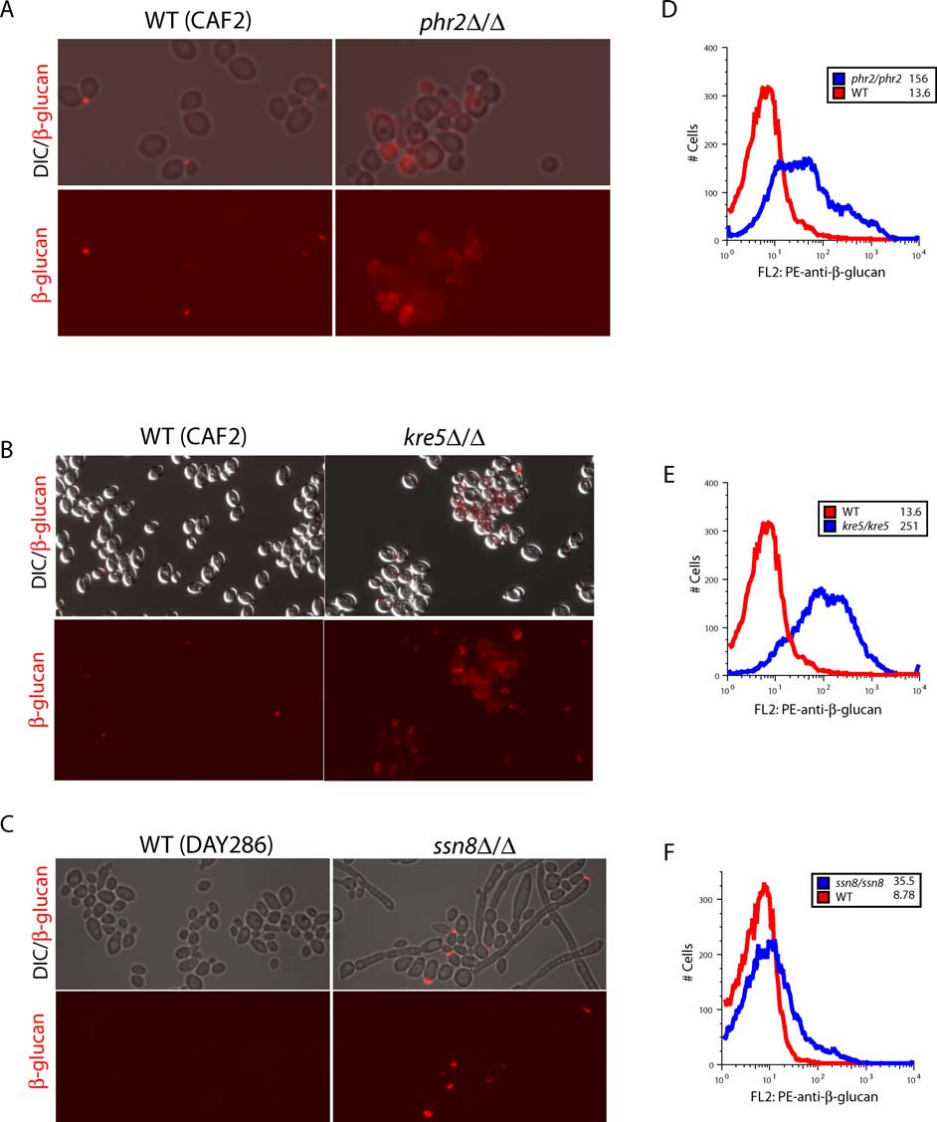
C. albicans Mutants of Masking Genes Have More Exposed β-Glucan Wild-type or mutant C. albicans strains were grown overnight in YPD, then stained with anti-β-glucan antibody and Cy3-labeled (A–C) or PE-labeled (D–F) secondary antibody. (A–C) Upper photomicrographs show overlay of brightfield and anti-β-glucan staining of Cy3-labeled cells; lower photomicrographs show anti-β-glucan staining alone. (D–F) Overlay histograms of FACS analysis of PE-labeled cells; data on 20,000 cells are shown. MFI values for wild-type and mutants are shown in insets. In parallel experiments, strains that were complemented with a wild-type copy of the gene showed full reversal of β-glucan exposure (for *KRE5*) or partial reduction in exposure (for *PHR2*) (Figure S5). This correlates with other phenotypes observed for these complemented strains [17,20,35].

The screen in S. cerevisiae identified potential genes and pathways regulating β-glucan masking in pathogenic fungi, and suggested that their C. albicans homologs might have a similar masking function in this pathogen. Mutations in two of these homologs *(PHR2* and *KRE5)* resulted in attenuated virulence in mice, and we reasoned that exposure of β-glucan may contribute to their reduced virulence. We found that mutations in each of three different β-glucan masking genes caused increased β-glucan exposure in C. albicans (Figure 5).

The *PHR2* gene is the only C. albicans homolog of the *S. cerevisiae GAS1* gene active under our growth conditions. It encodes a β-glucan transglycosylase required for β-glucan branching, cell wall integrity, and cell wall maintenance [17,18]. When grown in our conditions, the *phr2Δ/Δ* mutant displays a strong increase in the exposure of β-glucan (Figure 5A and 5D).

The homozygous *kre5Δ/Δ* mutant of C. albicans also showed a dramatic increase in β-glucan exposure (Figure 5B and 5E). We examined its function in C. albicans because it is nonessential in this fungus yet has a similar function to *KRE6,* which was identified in the S. cerevisiae screen (the *S. cerevisiae kre5Δ* mutant is lethal and therefore is not in the deletion library). Both *KRE5* and *KRE6* play important roles in the synthesis of β1,6-glucan, which is a minor component of the cell wall by weight but is important for cell wall organization and for anchoring of mannoproteins in the wall [19]. In *C. albicans,* homozygous *kre5Δ/Δ* mutants are avirulent in the mouse model of infection and have defects in filamentous growth and adherence to epithelial cells [20].

C. albicans has a single homolog of the S. cerevisiae gene, which encodes a global transcriptional regulator. The homozygous *ssn8Δ/Δ* mutant displays a mild increase in β-glucan exposure that is reproducibly found constrained to the tips of cells and filaments (Figure 5C and 5F). This polarized exposure of β-glucan is identical to that found in the phenotype of the *S. cerevisiae ssn8Δ* mutant, which is also altered in filamentation. The increased exposure of β-glucan at the tips and junctions, which are sites of cell wall remodeling, suggests that in wild-type growth there must be genes such as *SSN8* that direct the reconstitution of the wall. The homozygous *C. albicans ssn8Δ/Δ* mutant is mildly filamentous even in rich (yeast peptone dextrose [YPD]) media, which normally promotes yeast-form growth, showing that filamentation and β-glucan masking appear to be separable phenomena.

### Unmasked C. albicans Mutants Elicit More Proinflammatory Cytokines through the β-Glucan Receptor

**Figure 6:**
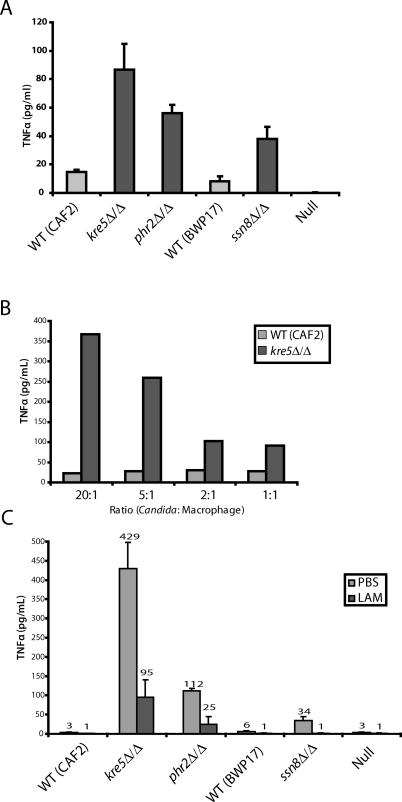
Unmasked C. albicans Mutants Hyperelicit TNFα from Macrophages through the β-Glucan Receptor (A) C. albicans strains were exposed to RAW264.7 macrophages at a ratio of 2:1 (yeast:macrophage), and supernatants were collected after 6 h. (B) Different numbers of C. albicans (wild-type or *kre5Δ*/*Δ* mutant) were exposed to BMDMs, and supernatants were collected after 6 h. (C) BMDMs were pretreated for 20 min on ice with media or soluble β-glucan (laminarin); they were then exposed to different C. albicans strains at a ratio of 10:1 (yeast:macrophage). After unbound fungi were washed off, macrophages were incubated for 6 h at 37 °C, and supernatants were collected for TNFα quantitation.

To test whether increased exposure of *Candida* β-glucan in these mutants also leads to altered immune recognition, we exposed wild-type or mutant *Candida* to RAW 264.7 macrophages and examined the elicitation of TNFα. As shown in Figure 6A, the *Candida* mutants with increased β-glucan exposure elicited higher levels of TNFα from macrophages. The relationship between β-glucan exposure and increased proinflammatory response is biologically relevant because unmasked mutants of S. cerevisiae (Figure S3) and C. albicans (Figure 6B) also elicited higher levels of TNFα and interleukin 6 (IL-6) (Figure S4) from BMDMs. This difference in elicitation is not simply a dosage effect, because this phenomenon occurs at different ratios of fungi to macrophages (Figure 6B). The altered signaling is also dependent on the β-glucan receptor, because the great majority of the increased elicitation of proinflammatory cytokines could be blocked by preincubation of the macrophages with the soluble β-glucan laminarin (Figure 6C). These data suggest that the majority (if not all) of the increased elicitation of proinflammatory cytokines occurs through the β-glucan receptor.

## Discussion

We found that “unmasking” the inner β-glucan layer of fungi causes their altered recognition by innate immune cells. Using the genome knockout library of S. cerevisiae as a guide, we identified the set of genes that establish and maintain the cell wall architecture in such a way that the majority of β-glucan is not recognized. The mutants identified in this screen are recognized better than the wild-type by the Dectin-1 β-glucan receptor, and they hyperelicit the proinflammatory cytokine TNFα from macrophages. Furthermore, mutation of cognate genes in the pathogen C. albicans leads to unmasking of β-glucan and hyperelicitation of proinflammatory cytokines in primary macrophages in a β-glucan-dependent manner. We also found that caspofungin unmasks fungal β-glucan at subinhibitory concentrations of the drug and alters recognition of C. albicans. This unanticipated activity may assist in fungal clearance during treatment of an infection.

The fungal mutants with exposed β-glucan and increased binding to Dectin-1 stimulate increased levels of proinflammatory cytokines independent of their bulk level of β-glucan. By creating barriers on their surfaces, fungal pathogens may mask certain PAMPs from the immune system and subvert the immune response. The immune system may also be able to counteract this fungal defense by unmasking the signature components of the fungus during the course of infection. For example, soluble or phagosomal proteases could remove the mannoprotein coat, which is known to expose β-glucan [21]. Differential exposure and unmasking could in part explain the diverse potential of fungi for pathogenesis.

The architecture of the fungal cell wall must be taken into account when assessing how fungi are detected by immune cells. The relative accessibility of β-glucan versus mannan on the surface could favor quite different responses from immune cells [22]. In this light, the finding that heat inactivation alters the cell wall to expose β-glucan suggests that experiments using heat-killed fungi or zymosan may benefit from complementary studies using live and/or UV-inactivated fungi.

The exposure of β-glucan on the cell surface might be altered by environmental conditions that affect the regulation of the genes we have shown to affect cell wall architecture. This effect could explain the reported greater binding of Dectin-1 to the yeast form of C. albicans as compared with its hyphal form [11]. In those experiments the fungus was grown on one medium to foster growth of the yeast form, and a different medium to induce the hyphal form. The different media could affect β-glucan masking independently of fungal morphotype [23]. Consistent with this view, preliminary results with C. albicans mutants locked in the yeast form suggest that simply modifying the media is sufficient to change the level of β-glucan masking (unpublished data). As all of our comparisons were done with strains grown on the same medium, the unmasking we observed cannot be explained by alterations in environmental conditions. Although yeast and hyphal forms showed no fundamental differences with respect to β-glucan accessibility and CF-induced unmasking, the morphotypes have other cell wall differences that result in differential immune recognition [22,24].

The weak proinflammatory response elicited by wild-type cells of C. albicans may prevent recruitment of effector cells and clearance of the fungus, and explain its persistence commensally in the gut in a large percentage of the population [25]. Both TNFα and IL-6 have been shown to be protective in the mouse model of disseminated candidiasis, suggesting that unmasking β-glucan could provoke a proinflammatory response and attenuate virulence in disseminated disease [26]. It is noteworthy that two of the unmasked C. albicans mutants (*phr2Δ/Δ* and *kre5Δ/Δ*) are avirulent [17,20], and it is possible that β-glucan unmasking contributes to their attenuated virulence. However, these mutations result in additional phenotypes [17,20] that cannot be excluded as a cause of their virulence defects. Evaluating the relevance of β-glucan masking to the immune response will require further experiments in an informative whole-animal model.

The finding that β-glucan unmasking by CF promotes the proinflammatory response suggests that antimycotics such as CF may strike at fungi in a dual way, killing them at high concentrations and unmasking their β-glucan at lower concentrations. In addition to enhancing nonopsonic, β-glucan-mediated uptake and signaling, drug-induced exposure of β-glucan may also target fungi for recognition by natural anti-β-glucan antibodies, which have been characterized in mouse and human sera [27]. Many other fungal pathogens are sensitive to CF, have homologs of β-glucan-masking genes, and can be sensed by Dectin-1 and/or targeted via their β-glucan [6,8,28,29]. The proteins encoded by these β-glucan-masking genes may provide useful drug targets for broad-spectrum treatment of fungal infection.

## Materials and Methods

### Fungal strains and growth.

S. cerevisiae strains in the BY4741 or BY4742 background were used to make the complete deletion library [30]. S. cerevisiae knockout libraries were purchased from Open Biosystems (Huntsville, Alabama, United States). C. albicans strains were derived from clinical isolate SC5314 [31]. Strain details are shown in Table S3. Fungi were grown overnight in YPD rich medium for yeast-form growth and RPMI 1640 for hyphal growth. C. albicans was grown at 37 °C; S. cerevisiae was grown at 30 °C. For CF treatment, overnight cultures of C. albicans CAF2 were diluted 1:1,000 into fresh YPD or RPMI 1640 containing dilutions of CF (caspofungin acetate, Cancidas formulation, Merck, Whitehouse Station, New Jersey, United States) and grown overnight. MIC_50_ was 2.5 ng/ml. For viability, cells were stained with propidium iodide (Sigma, St. Louis, Missouri, United States).

### Inactivation of fungi for macrophage interaction experiments.

For UV inactivation, the equivalent of 2.5 × 10^7^ cells from a culture were washed and resuspended in 1 ml of PBS in a six-well plate. The fungi were exposed to four doses of 100,000 μjoules/cm^2^ in a CL-1000 UV-crosslinker (UVP, Upland, California, United States), with agitation between each dose to treat cells evenly. For heat inactivation, 2.5 × 10^7^ cells in 1 ml of PBS were boiled for 10 min. After UV- or heat inactivation, cells were washed and renormalized by OD_600_. In *S. cerevisiae,* we found greater β-glucan exposure on cells that were heat-treated at 65 °C for 15 min, fixed for 30 min on ice in 3.7% formaldehyde, or fixed for 30 min on ice in 70% ethanol (unpublished data).

### Screen for β-glucan exposure.

Overnight cultures of strains were grown in YPD and stained with anti-β-glucan primary antibody (Biosupplies Inc., Parkville, Australia) and Cy3-labeled goat-anti-mouse secondary. This antibody is specific for β1,3-glucan [10], and costaining with this antibody and the purified Dectin-CRD shows colocalization (unpublished data). Stained cells were adhered to clear-bottom plates with concanavalin A (Sigma) and scanned with Cellomics VTI fluorescence microscopic imager (Cellomics, Pittsburgh, Pennsylvania, United States) using Target Acquisition software (Zeiss, Oberkochen, Germany). Mean average intensity and standard deviation of average intensity measurements were used to identify strains with over two standard deviations greater β-glucan exposure. Initially matA mutant strains were screened, then the independently constructed matα counterparts were screened. Dectin-CRD was expressed from pTrcHis2 vector (Invitrogen, Carlsbad, California, United States), purified from E. coli as described [11], and labeled directly with Alexa Fluor 555-succinimidyl ester (Molecular Probes, Eugene, Oregon, United States). Fungi were labeled with pure protein and showed glucan-inhibitable binding and similar staining pattern to that described [11].

### Macrophages.

RAW264.7 macrophages (ATCC, Manassas, Virginia, United States) were cultured in RPMI-10 (RPMI 1640 with 10% heat-inactivated fetal bovine serum and standard concentrations of penicillin-streptomycin; GIBCO, San Diego, California, United States). Cells were collected and plated in 24-well plates at 5 × 10^5^ cells/well. BMDMs were differentiated as described [32]. Briefly, bone marrow from BL/6 mice was cultured in RPMI-10 with 15% L-cell conditioned media and 25 ng/ml recombinant M-CSF (R&D Systems, Minneapolis, Minnesota, United States). Cells were collected after 6 d and plated in 24-well plates at 5 × 10^5^ cells/well. Mice were cared for in the WIBR animal facility under protocol # 0203–008–06.

### Macrophage-yeast interaction.

Fungi were grown overnight, washed extensively to remove any shed proteins or polysaccharides, and normalized by OD_600_ or hemacytometer (these two methods produced equivalent results). For *S. cerevisiae,* live fungi were added to RAW264.7 macrophages at equivalent effector:target (E:T) ratio of 1:5 in 24-well plates and incubated for 6 h, when supernatants were collected for ELISA. For exposure of *C. albicans,* UV-inactivated fungi were exposed either to RAW264.7 macrophages at an equivalent E:T of 1:2 or to BMDMs at an E:T of 1:10. For RAW264.7 exposures, supernatants were collected after 2 h; for BMDM exposures, supernatants were collected after 6 h. For blocking, BMDMs were preincubated with 100 μg/ml laminarin for 20 min on ice, exposed to fungi for 30 min at 37 °C, washed extensively, then incubated for 6 h more at 37 °C. ELISAs were performed according to the manufacturer's instructions (R&D Systems). Note that soluble β-glucan (laminarin) alone does not stimulate the macrophages (Figure 6C).

### Staining, microscopy, and FACS.

Fungi were grown overnight, washed, blocked in PBS + 2% BSA, and stained with either the anti-β-glucan antibody (followed by Cy3- or PE-labeled secondary antibody), Alexa Fluor-labeled Dectin-CRD, or Dectin-CRD-anti-Myc. Staining with antibody and Dectin-CRD is described above for the screen. For staining with Dectin-CRD-anti-Myc, Dectin-CRD was preincubated with FITC-labeled anti-Myc (Invitrogen) at a ratio of 3.4 μg of Dectin-CRD + 10 μl of anti-Myc, and then added to fungi at 1:100 on ice. Preincubation of Dectin-CRD with anti-Myc creates a probe with the same approximate size as the anti-β-glucan antibody but with the specificity of Dectin-1. Images of stained cells were taken using a Nikon TE2000-S microscope (Nikon, Tokyo, Japan) equipped with Spot RT camera (Diagnostic Instruments, Sterling Heights, Michigan, United States) and processed in Photoshop (Adobe Systems, Palo Alto, California, United States). Fluorescence was quantified on a FACScalibur cytometer (Becton-Dickinson, Palo Alto, California, United States); cells were gated by forward and side scatter based on wild-type cell size and shape, and mean fluorescence intensity of 20,000 labeled cells was calculated using Cellquest software (Becton-Dickinson).

## Supporting Information

Figure S1 Heat Inactivation of C. albicans Causes Greater β-Glucan Exposure and Greater Elicitation of TNFα Wild-type (CAF2) fungi were grown overnight in YPD medium at 37 °C. Cells were killed by UV irradiation or by heat inactivation (10 min at 100 °C).
(A) Live or killed cells were probed with anti-β-glucan antibody and PE-labeled secondary antibody and with Alexa Fluor 488-labeled Dectin-CRD, then subjected to FACS analysis.
(B) UV- or heat-killed cells were then exposed to BMDMs at a ratio of 10:1 (yeast:macrophage), and supernatants were taken after 6 h for measurement of TNFα levels.
(271 KB PDF)

Figure S2 Overlap of β-Glucan Cell Wall Architecture Screen with Other Cell Wall-Related Screens We examined the intersection (A) between the genes required for β-glucan masking (shown in green) and those identified by any one of four other genome-wide cell wall-directed screens (shown in dark red). These included (B) a screen to find mutants with synthetic lethality with genes required for β1,3-glucan biosynthesis (shown in dark blue [1]), (C) a screen for mutants with altered sensitivity to caspofungin (shown in light blue [1]), (D) two screens for mutants with altered sensitivity to the chitin-binding drug calcofluor white (shown in orange [2]), and (E) a screen for mutants with altered sensitivity to the cell wall-directed K1 killer toxin (shown in purple [3]).
(489 KB PDF)

Figure S3 The Unmasked *S. cerevisiae gas1Δ* Mutant Hyperelicits TNFα from Primary Macrophages BMDMs were exposed to different S. cerevisiae strains at a ratio of 5:1 (yeast:macrophage). After fungi were added, macrophages were incubated for 6 h at 37 °C, and supernatants were collected for TNFα quantitation.
(505 KB PDF)

Figure S4 Unmasked C. albicans Mutants Hyperelicit IL-6 from Macrophages through the β-Glucan Receptor BMDMs were pretreated for 20 min on ice with medium or soluble β-glucan (laminarin), and were then exposed to different C. albicans strains at a ratio of 10:1 (yeast:macrophage). After unbound fungi were washed off, macrophages were incubated for 6 h at 37 °C, and supernatants were collected for IL-6 quantitation.
(527 KB PDF)

Figure S5 Complementation of C. albicans Mutations Partially or Fully Reverses β-Glucan Exposure Phenotype Wild-type or mutant C. albicans strains were grown overnight in YPD, then stained with anti-β-glucan antibody and PE-labeled secondary antibody. Overlay histograms of FACS analysis of PE-labeled cells; data on 20,000 cells is shown. MFI values for wild-type and mutants are shown in insets. The partial reduction in β-glucan exposure (for *PHR2*) (A) or full reversal (for *KRE5*) (B) correlates with other phenotypes observed for these complemented strains [1–3].
(582 KB PDF)

Table S1 Overall Phenotype of Hypereliciting Mutants (106 KB DOC)

Table S2 Characterization of Exposed Mutants That Do Not Hyperelicit Cytokines (70 KB DOC)

Table S3 Fungal Strains (66 KB DOC)

## Abbreviations

BMDM: bone marrow-derived macrophage
CF: caspofungin
ConA: concanavalin A
CRD: carbohydrate recognition domain
IL-6: interleukin 6
PAMP: pathogen-associated molecular pattern
TNFα: tumor necrosis factor alpha
UV: ultraviolet light
YPD: yeast peptone dextrose
