# Supplementary figures and images for: A Drug-Sensitive Genetic Network Masks Fungi from the Immune System
Source: PLoS Pathog. 2006 Apr 28;2(4):e35. doi: 10.1371/journal.ppat.0020035 (PMC1447670; doi:10.1371/journal.ppat.0020035)

A

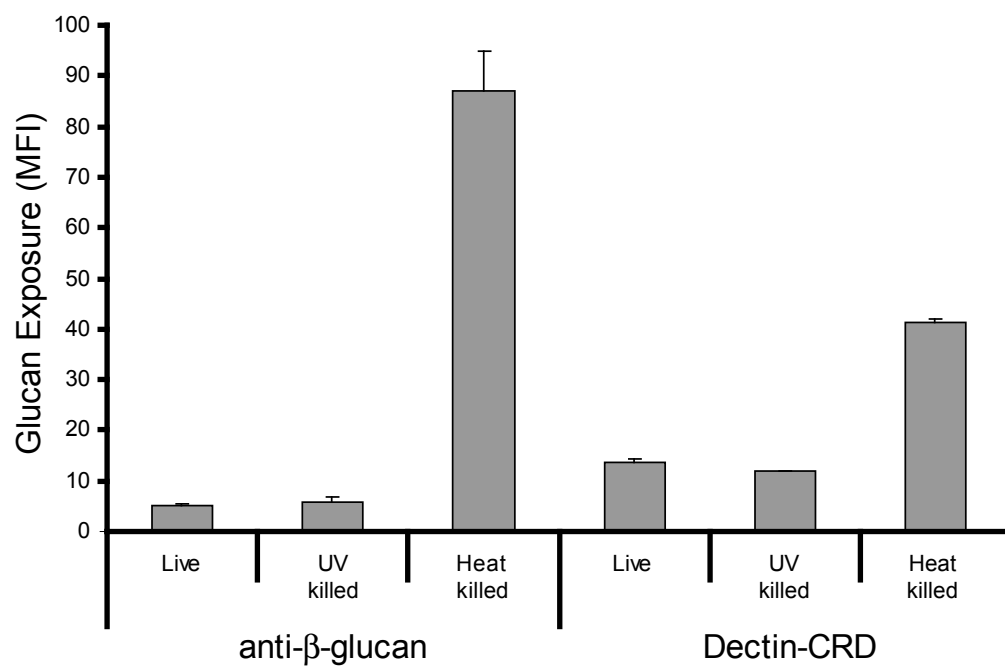

B

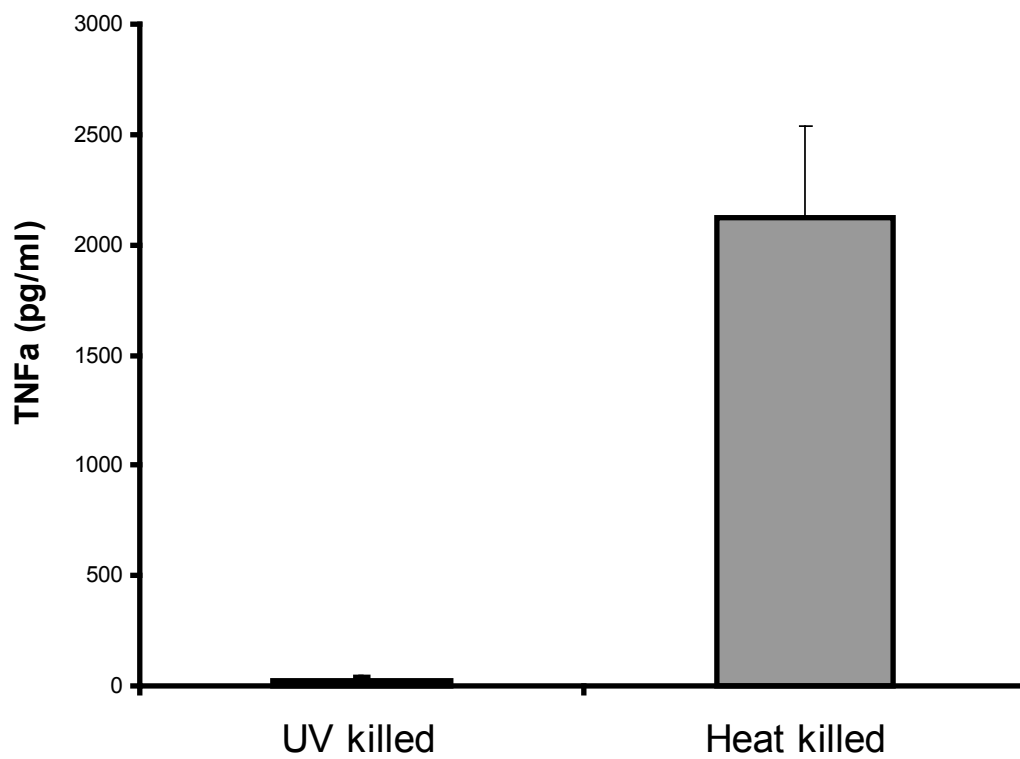

Supplement: Figure S1 — Wild-type (CAF2) fungi were grown overnight in YPD medium at 37 °C. Cells were killed by UV irradiation or by heat inactivation (10 min at 100 °C). (A) Live or killed cells were probed with anti-β-glucan antibody and PE-labeled secondary antibody and with Alexa Fluor 488-labeled Dectin-CRD, then subjected to FACS analysis. (B) UV- or heat-killed cells were then exposed to BMDMs at a ratio of 10:1 (yeast:macrophage), and supernatants were taken after 6 h for measurement of TNFα levels. (271 KB PDF) [file ppat.0020035.sg001.pdf]

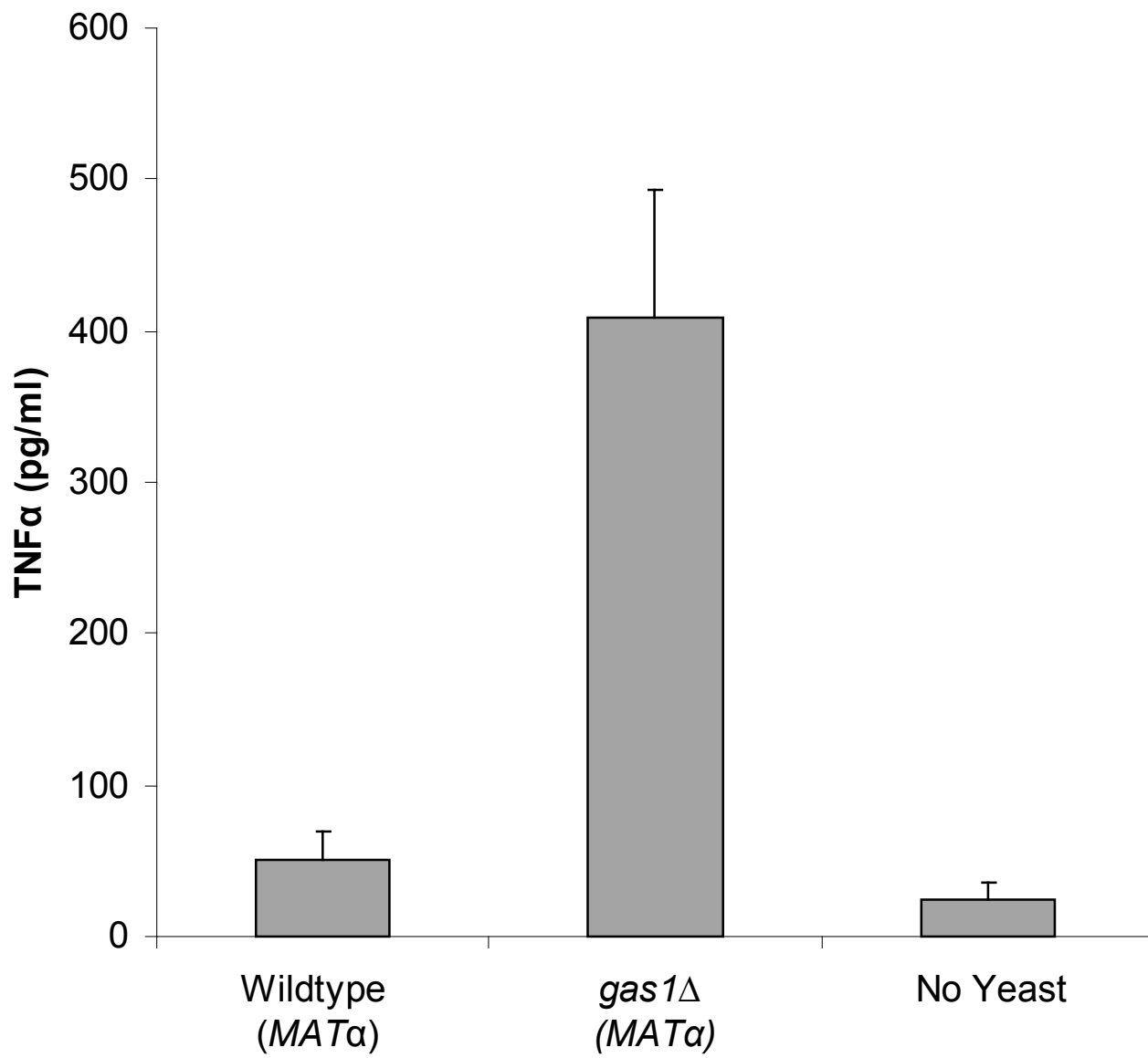

Supplement: Figure S3 — BMDMs were exposed to different S. cerevisiae strains at a ratio of 5:1 (yeast:macrophage). After fungi were added, macrophages were incubated for 6 h at 37 °C, and supernatants were collected for TNFα quantitation. (505 KB PDF) [file ppat.0020035.sg003.pdf]

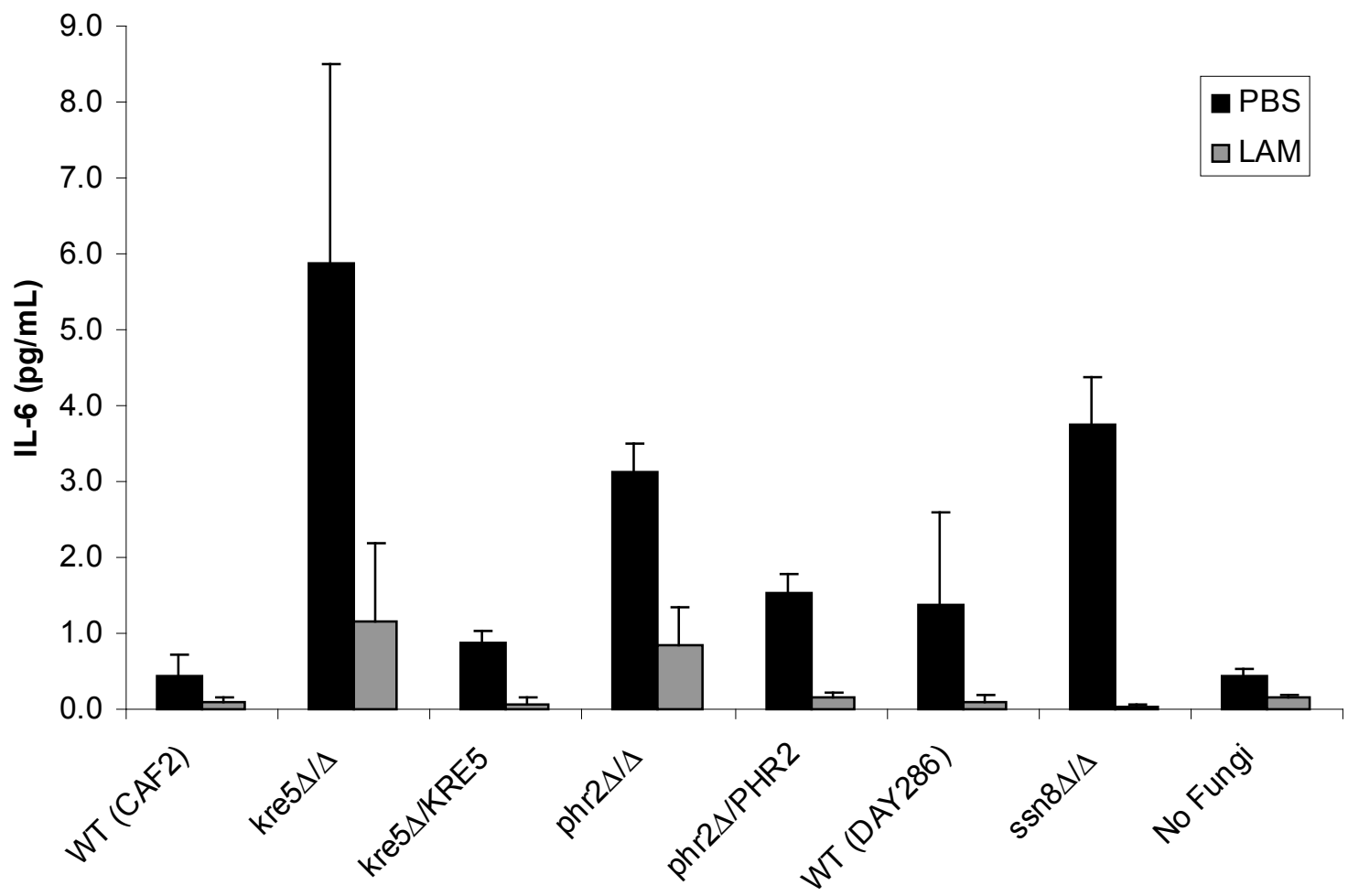

Supplement: Figure S4 — BMDMs were pretreated for 20 min on ice with medium or soluble β-glucan (laminarin), and were then exposed to different C. albicans strains at a ratio of 10:1 (yeast:macrophage). After unbound fungi were washed off, macrophages were incubated for 6 h at 37 °C, and supernatants were collected for IL-6 quantitation. (527 KB PDF) [file ppat.0020035.sg004.pdf]
