# Supplementary material for: A Drug-Sensitive Genetic Network Masks Fungi from the Immune System
Source: PLoS Pathog. 2006 Apr 28;2(4):e35. doi: 10.1371/journal.ppat.0020035 (PMC1447670; doi:10.1371/journal.ppat.0020035)
Supplement: Figure S2 — We examined the intersection (A) between the genes required for β-glucan masking (shown in green) and those identified by any one of four other genome-wide cell wall-directed screens (shown in dark red). These included (B) a screen to find mutants with synthetic lethality with genes required for β1,3-glucan biosynthesis (shown in dark blue [1]), (C) a screen for mutants with altered sensitivity to caspofungin (shown in light blue [1]), (D) two screens for mutants with altered sensitivity to the chitin-binding drug calcofluor white (shown in orange [2]), and (E) a screen for mutants with altered sensitivity to the cell wall-directed K1 killer toxin (shown in purple [3]). (489 KB PDF) [file ppat.0020035.sg002.pdf]

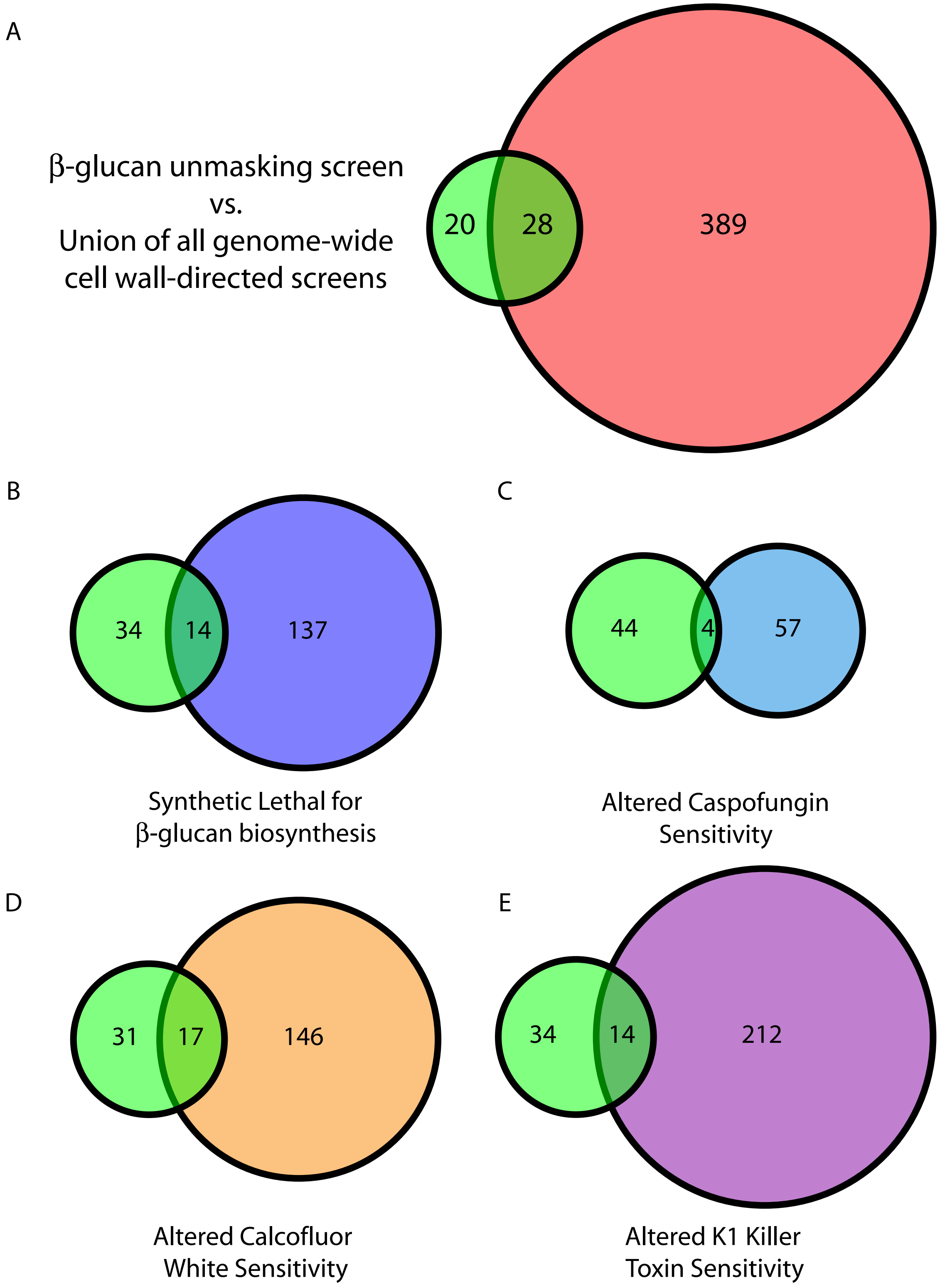

A
β-glucan unmasking screen
vs.
Union of all genome-wide
cell wall-directed screens
20
28
389
B
34
14
137
Synthetic Lethal for
β-glucan biosynthesis
C
44
4
57
Altered Caspofungin
Sensitivity
D
31
17
146
Altered Calcofluor
White Sensitivity
E
34
14
212
Altered K1 Killer
Toxin Sensitivity

References:

1. Lesage G, Sdicu AM, Menard P, Shapiro J, Hussein S, et al. (2004) Analysis of beta-1,3-glucan assembly in Saccharomyces cerevisiae using a synthetic interaction network and altered sensitivity to caspofungin. Genetics 167: 35-49.
2. Lussier M, White AM, Sheraton J, di Paolo T, Treadwell J, et al. (1997) Large scale identification of genes involved in cell surface biosynthesis and architecture in Saccharomyces cerevisiae. Genetics 147: 435-450.
3. Page N, Gerard-Vincent M, Menard P, Beaulieu M, Azuma M, et al. (2003) A Saccharomyces cerevisiae genome-wide mutant screen for altered sensitivity to K1 killer toxin. Genetics 163: 875-894.
