# Supplementary material for: A Drug-Sensitive Genetic Network Masks Fungi from the Immune System
Source: PLoS Pathog. 2006 Apr 28;2(4):e35. doi: 10.1371/journal.ppat.0020035 (PMC1447670; doi:10.1371/journal.ppat.0020035)
Supplement: Figure S5 — Wild-type or mutant C. albicans strains were grown overnight in YPD, then stained with anti-β-glucan antibody and PE-labeled secondary antibody. Overlay histograms of FACS analysis of PE-labeled cells; data on 20,000 cells is shown. MFI values for wild-type and mutants are shown in insets. The partial reduction in β-glucan exposure (for PHR2) (A) or full reversal (for KRE5) (B) correlates with other phenotypes observed for these complemented strains [1–3]. (582 KB PDF) [file ppat.0020035.sg005.pdf]

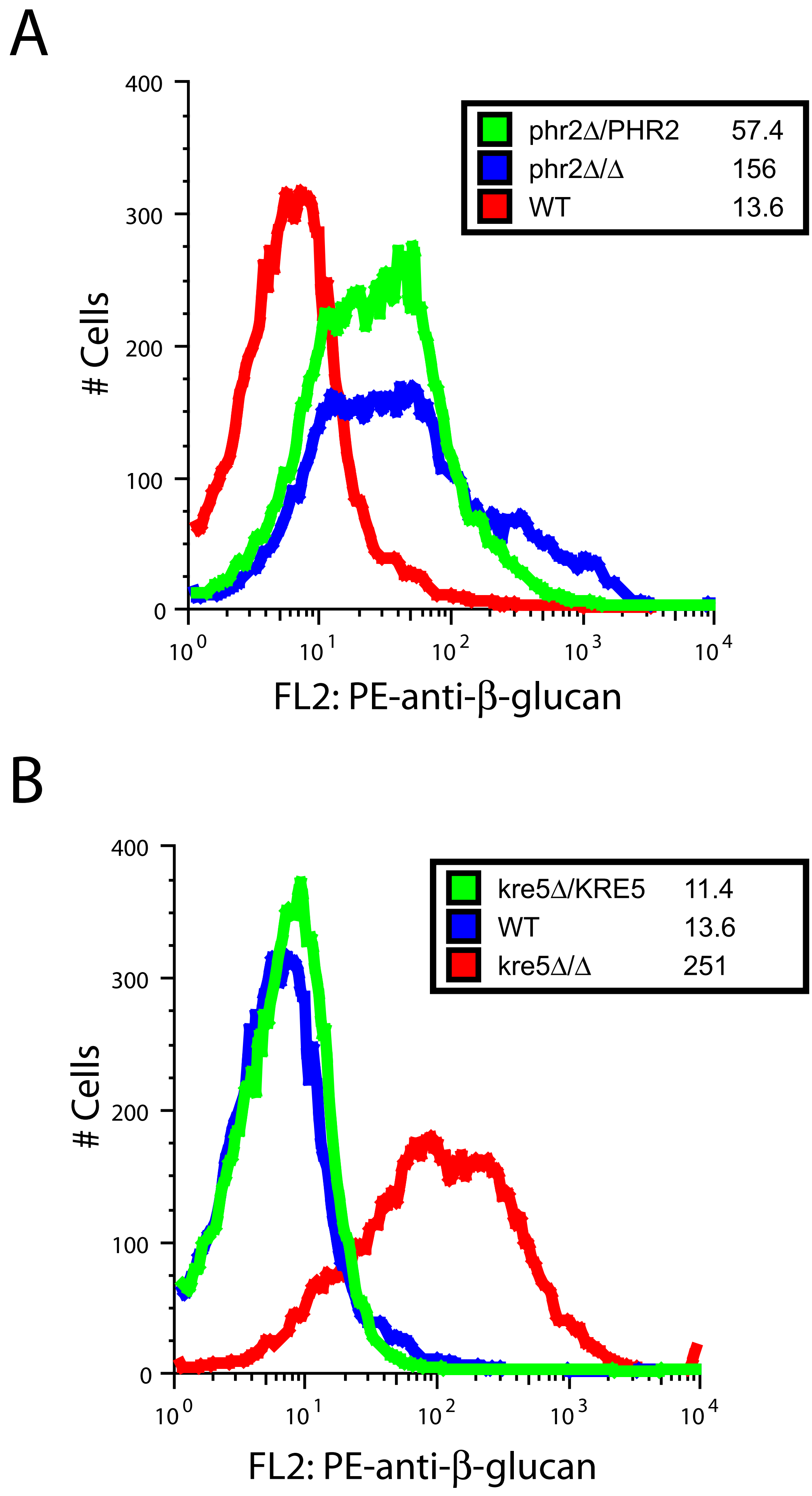
A
400
300
200
100
0
Cells
phr2Δ/PHR2 57.4
phr2Δ/Δ 156
WT 13.6
10 0
10 1
10 2
10 3
10 4
FL2: PE-anti-β-glucan
B
400
300
200
100
0
Cells
kre5Δ/KRE5 11.4
WT 13.6
kre5Δ/Δ 251
10 0
10 1
10 2
10 3
10 4
FL2: PE-anti-β-glucan

References:

1. Muhlschlegel FA, Fonzi WA (1997) PHR2 of Candida albicans encodes a functional homolog of the pH-regulated gene PHR1 with an inverted pattern of pH-dependent expression. Mol Cell Biol 17: 5960-5967.
2. De Bernardis F, Muhlschlegel FA, Cassone A, Fonzi WA (1998) The pH of the host niche controls gene expression in and virulence of Candida albicans. Infect Immun 66: 3317-3325.
3. Herrero AB, Magnelli P, Mansour MK, Levitz SM, Bussey H, et al. (2004) KRE5 gene null mutant strains of Candida albicans are avirulent and have altered cell wall composition and hypha formation properties. Eukaryot Cell 3: 1423-1432.
