# Supplementary material for: A Drug-Sensitive Genetic Network Masks Fungi from the Immune System
Source: PLoS Pathog. 2006 Apr 28;2(4):e35. doi: 10.1371/journal.ppat.0020035 (PMC1447670; doi:10.1371/journal.ppat.0020035)
Supplement: Table S1 — (106 KB DOC) [file ppat.0020035.st001.doc]

**Table S1: Overall phenotype of hyper-eliciting mutants**

| **YORF NUMBER** | **NAME** | **Proteome Database Description** | **ANTI-β-GLUCAN** | **DECTIN-CRD** | **TNFα** | **TNFα (%WT)** |
| --- | --- | --- | --- | --- | --- | --- |
| YLR131C | ACE2 | Metallothionein expression activator with similarity to Swi5p, has three tandem C2H2-type zinc fingers, required for delaying G1 phase specifically in daughter cells | 2 | 2 | 3 | 499 |
| YLR370C | ARC18 | Component of the ARP2/3 actin-organizing complex, involved in actin assembly and function | 2 | 3 | 3 | 447 |
| YLR242C | ARV1 | Protein involved in sterol uptake and distribution into the plasma membrane, required for normal sphingolipid metabolism | 2 | 1 | 1 | 164 |
| YJL115W | ASF1 | Anti-silencing function 1, a component of replication-coupling chromatin assembly factor (RCAF) | 1 | 2 | 1 | 207 |
| YBR200W | BEM1 | Protein required for cell polarization and bud formation | 1 | 1 | 2 | 360 |
| YPL161C | BEM4 | Bud emergence protein that interacts with Rho-type GTPases | 1 | 0 | 1 | 227 |
| YNL271C | BNI1 | Stimulates actin (Act1p) filament assembly and protects growing actin ends from excess capping protein, required for bipolar budding pattern | 1 | 1 | 2 | 286 |
| YNL298W | CLA4 | Serine/threonine protein kinase required for cytokinesis | 1 | 2 | 2 | 321 |
| YNL225C | CNM67 | Protein involved in nuclear migration and component of the spindle pole body | 2 | 3 | 3 | 734 |
| YDL117W | CYK3 | Protein involved in cytokinesis | 1 | 2 | 1 | 247 |
| YOR080W | DIA2 | Protein involved in invasive growth, contains an F-box, tetratricopeptide (TPR) repeats and leucine-rich (LRR) repeats | 2 | 1 | 2 | 280 |
| YBL047C | EDE1 | Protein with role in endocytosis | 2 | 2 | 3 | 421 |
| YNL084C | END3 | Protein required for endocytosis and cytoskeletal organization | 2 | 2 | 3 | 634 |
| YDR414C | ERD1 | Protein required for retention of luminal ER proteins | 2 | 1 | 2 | 301 |
| YFR019W | FAB1 | Phosphatidylinositol-3-phosphate 5-kinase involved in orientation or separation of mitotic chromosomes | 1 | 3 | 1 | 205 |
| YCR034W | FEN1 | Protein involved in the elongation of fatty acids up to 24 carbons | 2 | 2 | 1 | 217 |
| YMR307W | GAS1 | 1,3-beta-Glucanosyltransferase, glycophospholipid-anchored surface glycoprotein that regulates the crosslinking of beta-1,6-glucans in the cell wall | 2 | 1 | 2 | 281 |
| YER122C | GLO3 | GTPase-activating protein (GAP) for ADP-ribosylation factors Arf1p and Arf2p, involved in retrograde transport between Golgi and ER as well as endocytosis | 2 | 0 | 2 | 293 |
| YER014W | HEM14 | Protoporphyrinogen oxidase, converts protoporphyrinogen to protoporphyrin during heme biosynthesis | 2 | 1 | 2 | 198 |
| YMR032W | HOF1 | Homolog of cdc15 (S. pombe cdc15) 1, an SH3 domain-containing protein involved in cytokinesis, required for formation of a novel actin belt structure in the bud neck | 1 | 2 | 3 | 627 |
| YEL044W | IES6 | Ino Eighty Subunit 6 | 1 | 2 | 1 | 212 |
| YDR532C | KRE28 | Protein of the spindle pole body, forms a complex with Spc105p | 1 | 1 | 2 | 365 |
| YPR159W | KRE6 | Glucan synthase subunit required for synthesis of beta-1,6-glucan | 3 | 2 | 3 | 997 |
| YJL062W | LAS21 | Protein required for addition of a side chain to the glycosylphosphatidylinositol (GPI) core structure | 2 | 0 | 1 | 172 |
| YDR245W | MNN10 | Subunit of the M-Pol II mannosyltransferase complex | 3 | 2 | 3 | 1024 |
| YJL183W | MNN11 | Subunit of the M-Pol II mannosyltransferase complex | 3 | 2 | 3 | 1080 |
| YGL038C | OCH1 | Alpha-1,6-mannosyltransferase | 2 | 2 | 3 | 983 |
| YOR085W | OST3 | Oligosaccharyltransferase gamma subunit | 2 | 0 | 1 | 156 |
| YDL232W | OST4 | Oligosaccharyltransferase subunit | 3 | 1 | 3 | 425 |
| YCR044C | PER1 | Molecular function unknown | 3 | 1 | 1 | 201 |
| YPL133C | RDS2 | Protein with similarity to transcription factors | 1 | 1 | 1 | 140 |
| YNL069C | RPL16B | Ribosomal protein L16 | 2 | 2 | 1 | 174 |
| YCR009C | RVS161 | Protein required for viability after nitrogen, carbon, or sulfur starvation, also required for internalization step of endocytosis and for cell fusion during mating | 3 | 3 | 2 | 312 |
| YDR388W | RVS167 | Protein that affects actin distribution and bipolar budding | 3 | 3 | 2 | 383 |
| YDR159W | SAC3 | Nuclear protein required for mRNA export from the nucleus to the cytoplasm and for leucine transport, mutants exhibit defects in cytoskeletal function and in mitosis | 2 | 3 | 2 | 396 |
| YDR129C | SAC6 | Fimbrin, an actin filament bundling protein essential for polarized secretion | 3 | 3 | 1 | 243 |
| YBR171W | SEC66 | Component of ER protein-translocation subcomplex with Sec62p, Sec63p, Sec66p, and Sec72p | 1 | 1 | 3 | 458 |
| YER068W | MOT2 | Zinc finger transcriptional repressor, involved in G protein mediated pheromone signal transduction and member of the CCR4-Not complex | 2 | 3 | 2 | 273 |
| YBL007C | SLA1 | Protein involved in assembly of cortical actin cytoskeleton, has three SH3 domains | 3 | 3 | 3 | 676 |
| YHR030C | SLT2 | Serine-threonine protein kinase of the MAP kinase family involved in the cell wall integrity (low-osmolarity) pathway and in G2 phase cell-cycle checkpoint control | 1 | 1 | 2 | 369 |
| YNL025C | SSN8 | Cyclin C homolog, component of RNA polymerase holoenzyme complex and Kornberg's mediator (SRB) subcomplex | 1 | 2 | 1 | 246 |
| YAL016W | TPD3 | Regulatory A subunit of protein serine-threonine phosphatase 2A, activated by ceramide, required for normal shmoo formation and mating efficiency | 1 | 2 | 3 | 607 |
| YLR425W | TUS1 | GDP-GTP exchange factor for Rho1p, involved in the cell integrity signaling pathway | 2 | 3 | 1 | 249 |
| YLR386W | VAC14 | Protein involved in Fab1p-dependent phosphatidylinositol(3,5) bisphosphate synthesis | 2 | 3 | 2 | 290 |
| YLR337C | VRP1 | Proline-rich protein verprolin, involved in cytoskeletal organization and cellular growth | 3 | 3 | 3 | 1048 |
| YMR315W | YMR315W | Member of the oxidoreductase family NAD-binding Rossmann fold family, contains a GFO, IDH, or MOCA oxidoreductase C-terminal alpha or beta domain | 1 | 1 | 1 | 160 |
| YNL045W | YNL045W | Bifunctional leukotriene A4 hydrolase and anion-activated leucyl aminopeptidase | 1 | 0 | 1 | 157 |
| YPL158C | YPL158C | Protein of unknown function | 2 | 3 | 1 | 216 |

1) Mutants with significantly increased binding to anti-β−glucan antibody or Dectin-CRD were categorized into very high (3), high (2), moderate (1) or no (0) increase in staining.

2) Mutants with significantly increased elicitation of TNFα were categorized into very high (3), high (2), or moderate (1) increase in elicitation of TNFα from RAW264.7 macrophages

3) Average TNFα elicitation (expresses as percent of elicitation by wild-type control) over greater than three independent experiments. Standard deviations were all less than 20% of average.
