# Supplementary material for: A Drug-Sensitive Genetic Network Masks Fungi from the Immune System
Source: PLoS Pathog. 2006 Apr 28;2(4):e35. doi: 10.1371/journal.ppat.0020035 (PMC1447670; doi:10.1371/journal.ppat.0020035)
Supplement: Table S2 — (70 KB DOC) [file ppat.0020035.st002.doc]

**Table S2: Characterization of exposed mutants that do not hyper-elicit cytokines**

| **YORF #** | **NAME** | **Description** | **ConA*** | **CRD-MYC*** | **Dectin-CRD*** |
| --- | --- | --- | --- | --- | --- |
| YGL084C | GUP1 | Functions in glycerol uptake | 137 | 210 | 228 |
| YGR166W | KRE11 | Function in Golgi trafficking | 90 | 153 | 200 |
| YLR111W | YLR111W | Unknown function | 104 | 151 | 174 |
|  |  |  |  |  |  |
| YNL322C | KRE1 | Cell wall beta-glucan assembly | 92 | 287 | 140 |
| YJL139C | YUR1 | Probable glycosyltransferase | 101 | 187 | 131 |
| YAL023C | PMT2 | Functions in mannosylation | 104 | 184 | 136 |
| YAL058W | CNE1 | Functions in endoplasmic reticulum protein quality control | 94 | 178 | 120 |
| YGL027C | CWH41 | ER glucosidase | 103 | 152 | 137 |
|  |  |  |  |  |  |
| YLR110C | CCW12 | Cell wall mannoprotein | 91 | 139 | 225 |
| YOR026W | BUB3 | Required for cell cycle arrest upon loss of microtubule function | 108 | 112 | 212 |
| YGR188C | BUB1 | checkpoint kinase | 102 | 95 | 196 |
| YDR525W | API2 | Unknown function | 102 | 118 | 187 |
| YOR035C | SHE4 | Required for mother cell-specific HO expression | 88 | 70 | 185 |
| YGR167W | CLC1 | Clathrin light chain | 87 | 142 | 176 |
| YDR348C | YDR348C | Unknown function | 96 | 112 | 164 |
| YGL020C | GET1 | Role in Golgi protein sorting | 122 | 94 | 162 |
| YDR372C | VPS74 | Role in apical bud growth | 103 | 99 | 158 |
| YOR008C | SLG1 | Cell wall integrity and stress response component 1 | 92 | 112 | 157 |
| YML115C | VAN1 | Functions in mannosylation | 76 | 127 | 155 |
| YGL195W | GCN1 | Starvation signaling | 109 | 89 | 154 |
| YDL095W | PMT1 | Functions in mannosylation | 105 | 128 | 153 |
|  |  |  |  |  |  |
| YIR009W | MSL1 | Splicing factor | 150 | 86 | 148 |
| YDR126W | SWF1 | Involved in vacuolar trafficking | 99 | 81 | 138 |
| YDR207C | UME6 | Regulator of both repression and induction of meosis | 143 | 129 | 137 |
| YOL081W | IRA2 | Negatively regulates cAPK by antagonizing CDC25 | 99 | 113 | 126 |
| YBR015C | MNN2 | Functions in mannosylation | 77 | 101 | 116 |
| YOR115C | TRS33 | Functions in Golgi trafficking | 96 | 82 | 111 |
| YML019W | OST6 | Functions in mannosylation | 89 | 94 | 103 |
|  |  |  |  |  |  |
| *All values shown as percentage of wildtype levels. Values in red are >1.5x wildtype and values in green are significantly lower than wildtype. | | | | | |
