# Supplementary material for: A Drug-Sensitive Genetic Network Masks Fungi from the Immune System
Source: PLoS Pathog. 2006 Apr 28;2(4):e35. doi: 10.1371/journal.ppat.0020035 (PMC1447670; doi:10.1371/journal.ppat.0020035)
Supplement: Table S3 — (66 KB DOC) [file ppat.0020035.st003.doc]

**Table S3: Fungal strains**

| Strain Name | Genotype | Strain Background | Source | Reference | Species |
| --- | --- | --- | --- | --- | --- |
| BY4741 | MATa; *his3Δ1; leu2Δ0; met15Δ0; ura3Δ0* | S288c | ATCC | [3] | *S. cerevisiae* |
| BY4742 | matα *his3Δ1; leu2Δ0; lys2Δ0; ura3Δ0* | S288c | ATCC | [3] | *S. cerevisiae* |
| Deletions | BY4741 or BY4742 *orfX::kanMX6* | S288c | ATCC | [3] | *S. cerevisiae* |
| CAF2 | SC5314 *ura3::imm434* | SC5314 | W. Fonzi | [4] | *C. albicans* |
| CFM2 | *ura3::imm34/ura3::imm434 Δphr2::hisG/Δphr2::hisG-URA3-hisG* | CAF2 | W. Fonzi | [1] | *C. albicans* |
| CFM3 | *Δura3::imm434/Δura3::imm434 Δphr2::hisG/PHR2* | CAF2 | W. Fonzi | [1] | *C. albicans* |
| KAH3 | *Δura3::imm434/Δura3::imm434 Δkre5::hisG/Δkre5::hisG-URA3-hisG* | CAF2 | C. Abeijon | [2] | *C. albicans* |
| KAH4 | *Δura3::imm434/Δura3::imm434 Δkre5::hisG/Δkre5::hisG* + pLC14KRE5-URA3 | CAF2 | C. Abeijon | [2] | *C. albicans* |
| BWP17 | *Δura3::imm434/Δura3::imm434 Δarg4::hisG/Δarg4::hisG Δhis1::hisG/Δhis1::hisG* | SC5314 | A. Mitchell | [5] | *C. albicans* |
| *ssn8Δ/Δ* | *Δura3::imm434/Δura3::imm434 Δarg4::hisG/Δarg4::hisG Δhis1::hisG/Δhis1::hisG Δssn8::URA3/Δssn8:ura3-ARG4-ura3* | BWP17 | A. Mitchell | [6] | *C. albicans* |

References

1. Muhlschlegel FA, Fonzi WA (1997) PHR2 of Candida albicans encodes a functional homolog of the pH-regulated gene PHR1 with an inverted pattern of pH-dependent expression. Mol Cell Biol 17: 5960-5967.

2. Herrero AB, Magnelli P, Mansour MK, Levitz SM, Bussey H, et al. (2004) KRE5 gene null mutant strains of Candida albicans are avirulent and have altered cell wall composition and hypha formation properties. Eukaryot Cell 3: 1423-1432.

3. Winzeler EA, Shoemaker DD, Astromoff A, Liang H, Anderson K, et al. (1999) Functional characterization of the S. cerevisiae genome by gene deletion and parallel analysis. Science 285: 901-906.

4. Fonzi WA, Irwin MY (1993) Isogenic strain construction and gene mapping in Candida albicans. Genetics 134: 717-728.

5. Wilson RB, Davis D, Mitchell AP (1999) Rapid hypothesis testing with Candida albicans through gene disruption with short homology regions. J Bacteriol 181: 1868-1874.

6. Nobile CJ, Bruno VM, Richard ML, Davis DA, Mitchell AP (2003) Genetic control of chlamydospore formation in Candida albicans. Microbiology 149: 3629-3637.
